# Characterization of Peste des Petits Ruminants Virus, Eritrea, 2002–2011

**DOI:** 10.3201/eid1901.121072

**Published:** 2013-01

**Authors:** Gian Mario Cosseddu, Chiara Pinoni, Andrea Polci, Tesfaalem Sebhatu, Rossella Lelli, Federica Monaco

**Affiliations:** Author affiliations: Istituto “G. Caporale,” Teramo, Italy (G.M. Cosseddu, C. Pinoni, A. Polci, R. Lelli, F. Monaco);; National Animal and Plant Health Laboratory, Asmara, Eritrea (T. Sebhatu)

**Keywords:** peste des petits ruminants, peste des petits ruminants virus, ruminants, outbreaks, lineage IV, genetic characterization, family Paramyxoviridae, genus Morbillivirus, Eritrea, viruses

**To the Editor:** Peste des petits rumniants (PPR) is a highly contagious viral disease causing high rates of mortality among domestic and wild small ruminants in Africa and Asia. The disease causes economic losses in the countries where it is present. Peste des petits ruminants virus (PPRV) is a negative-sense, single-stranded RNA virus of the genus *Morbillivirus*, in the family *Paramyxoviridae*. Analysis of a small sequence of the PPRV nucleoprotein (NP) gene permits classification of the strains of the unique serotype of circulating PPRV into 4 genetically distinct lineages (*1*–*3*). The geographic distribution of lineages I and II is restricted mainly to western and central Africa and that of lineage III mainly to eastern Africa. Lineage IV is more widely distributed in Southeast Asia, the Arabian Peninsula, and the Middle East. Lineage IV is also currently circulating across northern and central Africa (*1*).

Although PPR is endemic in Eritrea, there are no data on the molecular characterization of the circulating viruses. As part of a program of cooperation between the National Animal and Plant Health Laboratory of Asmara and the Istituto Zooprofilattico Sperimentale dell’Abruzzo e Molise “G. Caporale” in Teramo, we analyzed 41 sheep and goat tissue samples that were collected by the Eritrean veterinary service during several outbreaks of PPR. Samples were collected in the following villages: 5 samples in Gahitelay (Northern Red Sea region) in 2003; 4 in Gulee and Weki (Maekel region), 3 in Hukum (Anseba region), and 6 in Torat and Keih Adi (Debub region) in 2005; and 6 in May Harish (Debub region) in 2011. For 17 samples, the region was not recorded; instead, they were identified as “Eritrea,” followed by the year of collection. Of the 41 samples, 22 were from goats and 6 from sheep; the source was not recorded for the other 13 samples. Nineteen samples were collected from lymph nodes, 10 from spleen, 9 from lung, and 1 each from tonsil, liver, and trachea. Samples were analyzed in the biosafety level 3 laboratory at the Istituto Zooprofilattico Sperimentale dell’Abruzzo e Molise.

Homogenates were prepared by using a mortar and sterile quartz to grind the tissue samples. The homogenates were then diluted (10% wt/vol) in phosphate-buffered saline, and tissue debris was removed by low-speed centrifugation. For lineage determination, total RNA was extracted by using the High Pure Viral Nucleic Acid Kit (Roche Diagnostic, Mannheim, Germany), RNA was amplified by reverse transcription PCR using the primer set NP3/NP4 (*4*) and the QIAGEN One-Step RT-PCR Kit (QIAGEN, Hilden, Germany). After agarose gel electrophoresis, 34 samples showed specific amplification of the 351-nt fragment of NP gene. PCR products were then purified by using the QIAquick PCR Purification Kit (QIAGEN) and sequenced by using the ABI PRISM Big Dye Terminator v3.1 Cycle Sequencing Kit (Applied Biosystems, Foster City, CA, USA) according to the manufacturer’s instructions.

Nucleotide sequences were obtained for 24 (59%) samples. The geographic distribution of animals in Eritrea with tissue samples from which we obtained viral sequences is shown in the Technical Appendix. Sequence editing, assembly, and alignment were performed by using BioEdit version 7.0.5.3 (*5*). BLAST (www.ncbi.nlm.nih.gov) was used to find homologous hits in the sequence databases. Phylogenetic analysis (neighbor-joining) with bootstrap (1,000 replicates) was performed using MEGA4 (*6*). We performed phylogenetic analysis of a 255-nt sequence of PPRV NP gene, using a selection of reference strains: 7 new sequences from this study (GenBank accession nos. JX398126, JX398127, JX398128, JX398129, JX398130, JX398131, JX398132) and 34 sequences representing the 4 lineages of PPRV for which sequences are available (Figure). Phylogenetic analysis was repeated by using the distance (neighbor-joining) method in PHYLIP version 3.67 (http://evolution.gs.washington.edu/phylip.html). The results of the phylogenetic analyses showed that the PPRV we isolated from ruminants in Eritrea belongs to the Afro-Asian lineage 4 and could be further distinguished into 2 clusters. 

**Figure:**
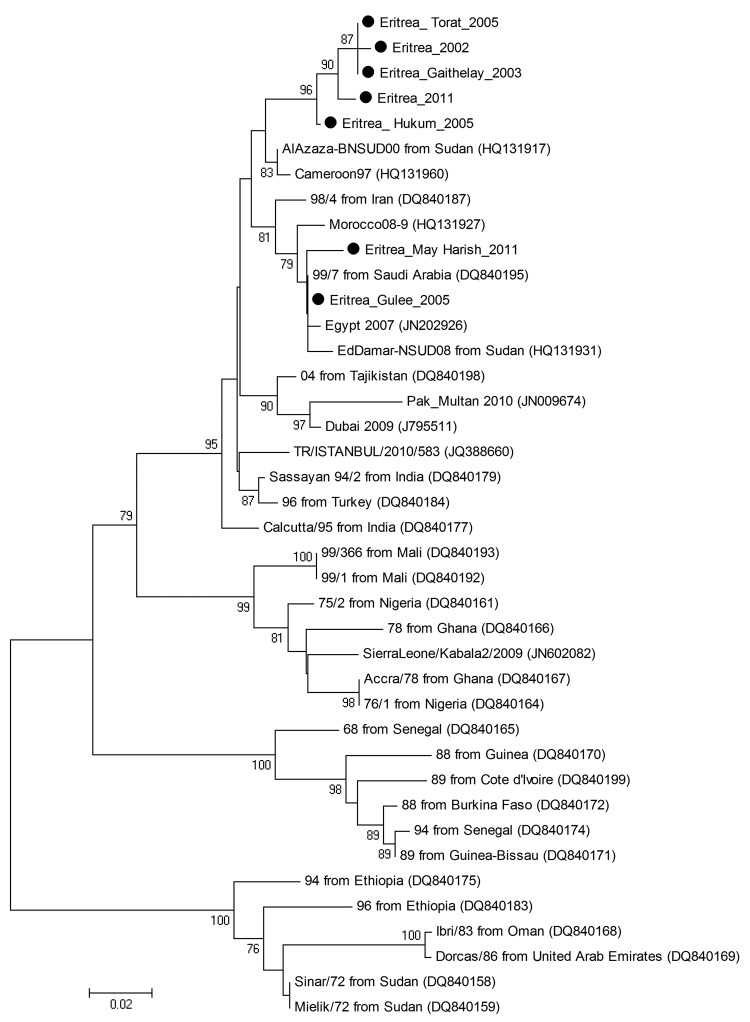
Phylogenetic tree showing the genetic relationships between isolates of peste des petits ruminants virus (PPRV). The tree was constructed on the basis of 255-nt sequences of the PPRV nucleoprotein gene. Black dots indicate sequences obtained in this study. Lineages are indicated on the right, and GenBank accession numbers are shown in parentheses. Analysis was performed by using the MEGA4 software (*6*) and neighbor-joining (maximum composite likelihood) methods. Bootstrap support values >70 are shown at nodes (1,000 replicates). The scale bar indicates nucleotide substitutions per site.

The first cluster consisted of 5 PPRV strains (Eritrea_2002, Eritrea_Gahitelay_2003, Eritrea_Hukum_2005, Eritrea_Torat_2005, and Eritrea_2011), which are related to PPRV strains from Sudan (AlAzaza_BNSUD00) and Cameroon (Cameroon_97). Thus, the data suggest that these 5 isolates from Eritrea are related to viruses that originated in Sudan and Cameroon and spread across central Africa, probably by free ranging wild or susceptible domestic animals.

The second cluster consisted of 2 PPRV strains (Eritrea_Gulee_2005 and Eritrea_May Harish_2011); this cluster also includes strains from Saudi Arabia (Saudi Arabia 99_7), Sudan (NSUD08), and Egypt (Egypt_2011). Genetic sequences are highly conserved in this group, particularly for Eritrea_Gulee_2005, which shares 100.0% nucleotide identity with Saudi Arabia 99_7, 99.6% identity with Egypt_2011, and 99.2% identity with NSUD08. The group is closely related to viruses collected in Morocco during outbreaks in 2008. The data suggest a clonal origin of the viruses belonging to this group, supporting the hypothesis that Eritrea could have been a gateway for the Saudi Arabia 99_7 strain to spread throughout Africa.

Our findings show that PPRV lineage IV is the dominant lineage circulating among ruminants in Eritrea. This information is crucial for further research aimed at defining strategies for the efficient prevention and control of PPR in this country.

Technical Appendix Distribution of ruminants in Eritrea with peste des petits ruminants virus infection, 2003–2011. Colored circles indicate regions from which tissue samples were collected from goats and sheep during outbreaks of peste des petits ruminants; nucleotide sequences for the samples were determined and analyzed.
