# Supplementary material for: Characterization of Peste des Petits Ruminants Virus, Eritrea, 2002–2011
Source: Emerg Infect Dis. 2013 Jan;19(1):160–1. doi: 10.3201/eid1901.121072 (PMC3558003; doi:10.3201/eid1901.121072)
Supplement: Technical Appendix — Distribution of ruminants in Eritrea with peste des petits ruminants virus infection, 2003–2011. Colored circles indicate regions from which tissue samples were collected from goats and sheep during outbreaks of peste des petits ruminants; nucleotide sequences for the samples were determined and analyzed. [file 12-1072-Techapp-s1.pdf]

Article DOI: http://dx.doi.org/10.3201/eid1901.121072

Characterization of Peste des Petits Ruminants Virus, Eritrea, 2002–2011

Technical Appendix

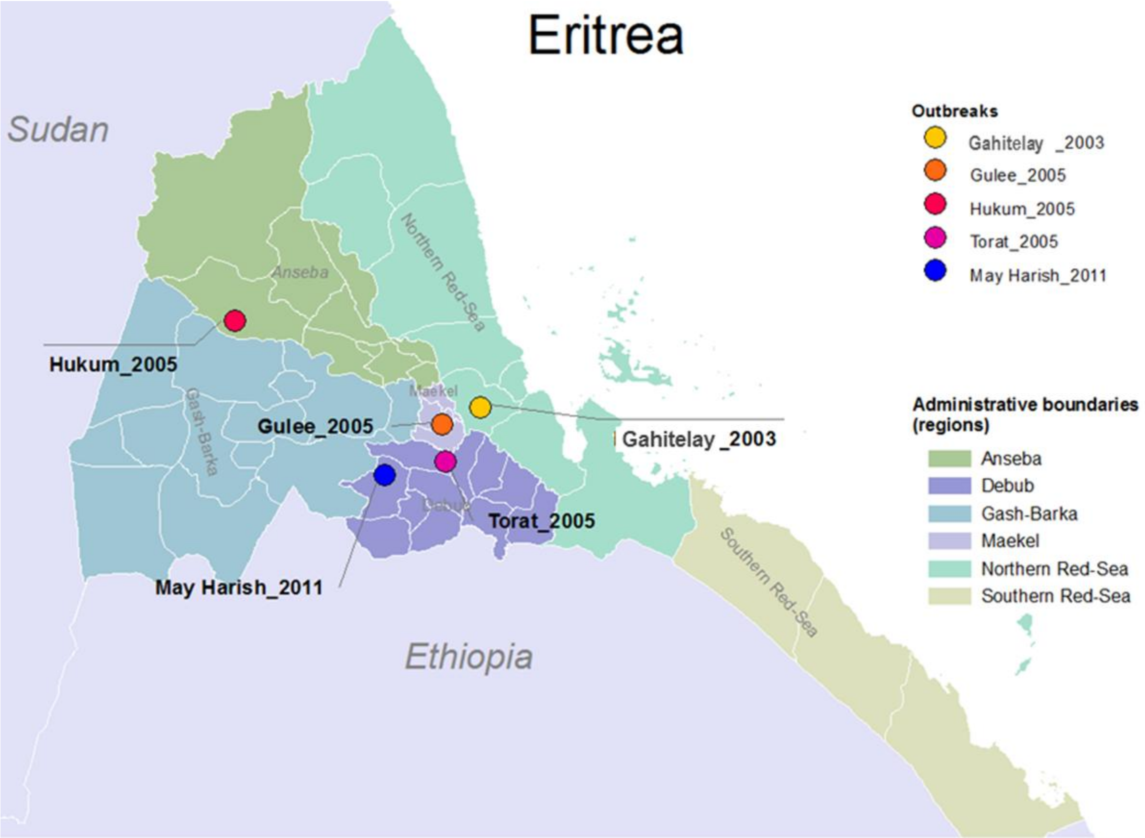

Figure. Distribution of ruminants in Eritrea with peste des petits ruminants virus infection, 2003–2011. Colored circles indicate regions from which tissue samples were collected from goats and sheep during outbreaks of peste des petits ruminants; nucleotide sequences for the samples were determined and analyzed.
